# Quantitative Trait Loci and Candidate Genes Associated with Cold-Acclimation and *Microdochium nivale* Tolerance/Susceptibility in Winter Triticale (x *Triticosecale*)

**DOI:** 10.3390/plants10122678

**Published:** 2021-12-06

**Authors:** Gabriela Gołębiowska, Mateusz Dyda, Katarzyna Wajdzik

**Affiliations:** Institute of Biology, Pedagogical University of Cracow, Podchorążych 2, 30-084 Kraków, Poland; mmdyda89@gmail.com (M.D.); kasia.wajdzik@gmail.com (K.W.)

**Keywords:** cold-hardening, crops, fungal pathogens, cross tolerance, QTLs, resistance proteins, photosynthesis

## Abstract

Tolerance to pink snow mold caused by *Microdochium nivale* appears after a cold-hardening period and it is an essential, genotype-dependent, complex quantitative trait for the wintering of triticale (x *Triticosecale*) and other cereals. Despite long-term studies, a marker for the selection of the tolerant genotypes is still insufficiently recognized. Chlorophyll fluorescence has been reported as a sensitive indicator of stress effects on photosynthesis and can be used to predict plant tolerance. In this study, the genomic regions (QTLs) associated with the level of winter triticale seedlings damage caused by *M. nivale* infection as well as photosynthesis quantum efficiency and chlorophyll *a* fluorescence parameters were identified in seedlings of mapping population of 89 doubled haploids lines (DHs) derived from F_1_ hybrid of cv. ‘Hewo’ and cv. ‘Magnat’ accompanied with the genetic map consisting of 20 linkage groups with a total map length 4997.4 cm. Independent experiments performed in controlled conditions revealed 13 regions identified by a composite interval mapping, located on 7A, 1B, 2B, 6B, 7B, 3R, 5R, and 6R linkage groups and related to the PI, PI_ABS_, TR_o_/CS, ABS/CS, ABS/CS_m_, ABS/RC, and Qy values as well as *M. nivale* tolerance T and susceptibility level P expressed by the seedling damage index. Additionally, candidate genes were in silico identified with the sequence position on wheat (2B and 7B) and rye (5R) chromosomes, where relevant QTL regions were found. The most important candidate genes indicated for *M. nivale* tolerance of cold-hardened triticale seedlings include those coding: sterol 3-beta-glucosyltransferase UGT80A2-like, transcription factor NAI1-like, and flavonol3-sulfotransferase-like proteins on chromosomes 2B and 5R.

## 1. Introduction

Triticale (*x Triticosecale* Wittm.) originates from a cross between wheat and rye [1,2]. Consequently, it contains wheat (AABB or AABBDD) and rye (RR) genomes, whereas the D genome is eliminated during the breeding process. This crop is well adapted to adverse environmental conditions [3,4]. It also has a higher tolerance than wheat and rye to many fungal diseases [4,5,6] and its grain contains a higher level of essential amino acids than wheat [7]. These qualities make triticale a promising cereal and genetic source for transferring genes, in particular, tolerance genes from rye to wheat [4,6,8,9].

Despite the natural cereal’s resistance to fungal pathogens, snow mold is still one of the most serious diseases in the *Poaceae* family [10,11,12,13,14]. *Microdochium nivale* (Fr. Samuels and Hallett) causing the pink snow mold is one of the most widespread and virulent fungal pathogens in moderate and cold climates [10,15,16]. The first symptoms of *M. nivale* infection appear on the leaves as small patches of mycelium [10,13,17]. It causes chlorosis, which leads to drying leaves and creating a compressed layer [10,14,18]. Moreover, *M. nivale* may cause a leaf blotch and stem rot. It also co-exists in a fungal complex resulting in fusarium ear blight disease [10,14,19]. As a result, it causes significant yield losses of cereals during the winter period. Tolerance against *M. nivale* infection is a complex quantitative trait, which is dependent on many genes and environmental factors [14,17,18,20,21]. The fungicides used for its control are not sufficiently safe and effective [19,22,23]. Thus, it is essential to identify the mechanisms of tolerance and then introduce associated trait/s into new cultivars.

Photosynthetic electron transport (PET) chain and the photosystem II (PSII) are factors that are involved in the induction of defence-related genes as well as cold and light-related genes [14,24,25,26]. Chlorophyll *a* (Chl-*a*) fluorescence has been reported as a sensitive indicator of stress effects on photosynthesis [27,28]. For this purpose, chlorophyll *a* fluorescence analysis is used to analyze biochemical and physiological changes in the photosynthetic apparatus under changing environmental conditions [29]. All chlorophyll *a* fluorescence data might be analyzed using the JIP test based on the theory of energy flow in thylakoid membranes [30,31,32]. Multiple studies of photosynthetic apparatus response to freezing indicated that the Chl-*a* measurements can be used to predict plant freezing tolerance, for example, in triticale [28,32].

Our previous studies on the winter triticale tolerance to the infection of *M. nivale* proved the indispensability of cold-hardening for induction of plant defence [13,14,17,18,20,21,33,34,35]. Moreover, after cold-hardening, plants acquired different levels of tolerance: seedlings of cv. ‘Magnat’ remained susceptible while the tolerance of cv. ‘Hewo’ seedlings increased during the low-temperature exposure [17,18,20,21]. After this study, a population of 89 DH lines was developed from F_1_ hybrid between those cultivars by the anther culture method [36]. The multiple *M. nivale* tolerance tests in controlled conditions enabled the identification of DH lines from this population with the transgression of tolerance or susceptibility in comparison to the parental cultivars. Similar to our results, other studies found cold-hardening induced the cereal defence responses to *M. nivale* infection, and the obtained tolerance levels were genotype-dependent [14,37,38,39].

Cogent and efficient markers for the selection of the snow-mold resistance or tolerance are needed for winter triticale improvement. Therefore, the present study aimed to identify quantitative trait loci (QTLs) associated with *M. nivale* tolerance/susceptibility evaluated in three seasons along with quantum efficiency of photosynthesis and Chl-*a* fluorescence parameters in triticale seedlings. The DH mapping population enabled the location of genome regions related to analyzed traits. Subsequently, all available wheat and rye genomes were screened in silico for candidate genes, whose sequence was found within the significant QTL regions associated with triticale cold-acclimation and *M. nivale* tolerance/susceptibility.

## 2. Materials and Methods

### 2.1. Plant Material

In the present study, the mapping population consisted of 89 winter triticale DH lines derived from F_1_ hybrid of cv. ‘Hewo’ (Strzelce Plant Breeding-IHAR Group Ltd., Łódzkie, Poland) and cv. ‘Magnat’ (DANKO Plant Breeders Ltd., Kraków, Poland) together with both parental cultivars. All experiments were performed under controlled laboratory conditions at the Institute of Plant Physiology Polish Academy of Science (IPP PAS) in Kraków, Poland.

Well-formed kernels were surface-sterilized with 96% ethanol for 3 min and then with a 25% commercial mixture of sodium hypochlorite and detergents (Domestos, Unilever Polska) for 15 min. Kernels were washed in sterile water, then grown for 2 days at 26 °C (day/night) in darkness in plastic Petri dishes on a watered filter paper. Healthy seedlings were planted in a sterile mixture of soil/turf substrate/sand (2/2/1, *v/v/v*) in multi-pots. For each genotype, six seedlings were grown in one row of multi-pot in 3 replicates (18 plants in total) in a randomized complete block design for unhardened, cold-hardened, and inoculated plants separately. One plant was considered to be one biological replicate. Initially, all seedlings were grown under optimal controlled conditions (21/16 °C day/night) for 7 days. On the 7th day, plants were supplemented with Hoagland and Arnon’s [40] sterile medium, and then, two-thirds of multi-pots were transferred to the pre-hardening conditions (12/12 °C day/night) for 14 days. The remaining one-third of seedlings considered as unhardened was continuously grown under optimal controlled conditions (21/16 °C day/night) for the next 14 days, until obtaining the same developmental stage as the seedlings after a complete hardening. In contrast, pre-hardened plants were moved to the cold-hardening conditions (4/4 °C day/night) for 28 days. Then the half of the cold-hardened seedlings were inoculated with *M. nivale* mycelium, while one-half of them remained non-inoculated. The plant material preparation was repeated in each year of the experiment as described above. The experiment scheme and plant development stages were previously described in detail [20,35].

### 2.2. Degree of the Seedlings Damage Caused by M. nivale Infection (P Index) Evaluation

The degree of the seedlings damage (P index) was tested in controlled conditions over three years period (2011–2013) and assigned as P_2011_, P_2012_, and P_2013_ for each year accordingly. After a pre-hardening and hardening period, cold-hardened seedlings were divided into two parts. One-half of the seedlings was inoculated with the fungal soil-borne mycelium derived from the monosporal isolate of *Microdochium nivale* No. 38z/5a/01. For that purpose, mycelium was spread around each seedling in equal portions. The second half of the plants remained not inoculated. Both inoculated and non-inoculated plants were covered with watered lignin and black plastic bags to imitate conditions under a snow cover, then grown in the cold chamber in the dark, at 4 °C (day/night) for 21 days. Subsequently, all plants were uncovered and grown under optimal conditions (21/16 °C day/night) for 21 days as described by Gołębiowska and Wędzony [20].

After this period, the seedling re-growth and their injury by the *M. nivale* infestation were evaluated as P index in the arbitrary scale based on visual observation according to Prończuk and Madej [41]: 0—no symptoms of disease; 1–25%; 2–50%; 3–75% of tissue with disease symptoms; 4—dead plant. The whole plant damage index (P index) was calculated according to Townsend and Heuberg’s equation: P = Σ(100·^●^n·v)/V·N [%], where n—number of plants with the respective damage level; v—damage level 0–4 as described above; V—a maximal damage level; N—the number of assessed plants. The P index test was repeated according to the same protocol during three years of the present experiments. The level of plant tolerance was calculated for each experiment as a 100% − P_index_ value.

### 2.3. Analysis of Chlorophyll a Fluorescence

Eighteen leaves (second in appearance) from separate seedlings of each genotype were analyzed for unhardened plants (21 days old) as well as for cold-hardened ones on the 28th day from the start of a cold-hardening period. Chlorophyll *a* fluorescence was in vivo measured using FluorCam 700 ST 664-009656 fluorometer FMS 2 (Hansatech). The 500 μmol [quantum] m^−2^s^−1^ PPFD actinic light was used for chlorophyll fluorescence excitation in PSII and the steady-state fluorescence yield (Fs) was stabilized according to Maxwell and Johnson [42]. The maximal fluorescence yield (Fm) was measured in leaves, dark-adapted for 15 min according to Lichtenthaler et al. [43]. The following parameters were evaluated according to Rapacz et al. [32]: (1) specific energy fluxes for single PSII reaction centers (RCs): absorbed energy flux (ABS/RC), trapped energy flux (TR_o_/RC), electron transport flux (ET_o_/RC) and dissipated energy flux (DI_o_/RC); (2) phenomenological energy fluxes calculated for the area of the photosynthetic sample (CS) at t = 0: absorbed energy flux per CS (ABS/CS), trapped energy flux per CS (TR_o_/CS), electron transport flux per CS (ET_o_/CS) and dissipated energy flux per CS (DI_o_/CS). Calculated PSII performance indexes and reaction center densities included performance index calculated on an absorption basis (PI_ABS_) and densities of QA^−^ reducing PSII reaction centers at t = 0 and t_max_ (time to reach maximum fluorescence F_m_), corresponding to RC/CS_o_ and RC/CS_m_, respectively. The maximum quantum yield of primary photochemistry at t = 0 (F_v_/F_m_) was calculated, were F_v_-variable fluorescence yield. The maximal quantum yield of PSII (Qy) was measured in the light according to Genty et al. [44].

### 2.4. QTL Mapping and Statistical Analysis

For the quantitative trait loci analysis, the genetic map of the winter triticale DH ‘Hewo’ x ‘Magnat’ lines population described by Tyrka et al. [45] was used. This 4997.4 cm map contains 20 linkage groups assigned to the A (7), B (7), and R (6) genomes with 842 DArT, 2647 SNP-DArT, and 50 SSR markers [45]. After plant phenotyping in present experiments, the Shapiro–Wilk test at *p* ≤ 0.05, as well as skewness and kurtosis, were calculated to study a normal distribution of analyzed traits. The analysis of variance (ANOVA) of phenotypic data and the Pearson’s linear correlation between all characteristics were determined using Statistica version 13.0 (StatSoft. Inc.).

Each QTL region was identified using windows QTLCartographer 2.5 software according to Wang et al. [46]. The composite interval mapping (CIM) method was used to obtain the QTL region associated with all measured traits. The threshold logarithm of the odds (LOD) scores was calculated using 1000 permutations and a 1 cm walk speed at significance *p* ≤ 0.05. QTL with an LOD score ≥ 3.0 was accepted. The 95% QTL confidence intervals were determined with 1-LOD interval defined by the left and right markers using WinQTL Cartographer 2.5 [47,48]. The percentage of the phenotypic variation covered by the QTL region was calculated with a single factor regression (R^2^). The favourable alleles were selected in each QTL effect, based on the additive (Add) effects at LOD peaks obtained by Windows QTL Cartographer 2.5, where positive effect referred to cv. ‘Hewo’ and negative to cv. ‘Magnat’. The common name of each locus within QTL effects identified in a close position in cm was given and contained *Q* as QTL; *UH* or *H* as an abbreviation for the unhardened or hardened plant, respectively; *tHM* as triticale ‘Hewo’ x ‘Magnat’ population; name of triticale chromosome and the number of QTL region on each chromosome.

### 2.5. The in Silico Location of Genes within the QTLs

For in silico location of candidate genes, DArT and e-SNP sequences of the flanking and maximal LOD peak markers of the significant QTLs were used. Sequences of the wheat and rye DArT clones were downloaded from the Diversity Arrays Technology webpage (https://www.diversityarrays.com/technology-and-resources/sequences/, accessed on 1 November 2021). Then the wheat and rye DArT and e-SNP sequences were used to query all available wheat and rye genome collections for the physical mapping using the BLAST tool of GrainGenes Blast Service beta (https://doi.org/10.1093/molbev/msz185, accessed on 1 November 2021). Genes localized on target physical wheat and rye regions were retrieved and annotated with the use of BLAST^®^ (https://blast.ncbi.nlm.nih.gov/Blast.cgi, accessed on 1 November 2021). The sequences producing significant alignments and the highest query cover were selected. Next, the function of candidate genes was deduced from the UniProt database by using Quick Go annotation.

## 3. Results

The plant genotype and treatment as well as the interaction between those independent factors had a significant influence on all studied traits (*p* ≤ 0.05). Therefore, QTLs were calculated for the mean data of each experiment separately. The results of the Shapiro–Wilk test together with skewness and kurtosis calculation indicated the normal distribution of traits values in every experiment (Table S1). A total of 19 significant QTL effects were identified with the CIM method for P_2011_, P_2012_, P_2013_, T_2011_, T_2012_, and T_2013_, as well as PI, PI_ABS_, TR_o_/CS, ABS/CS, ABS/CS_m_, ABS/RC, and Qy_2012_ traits of triticale seedlings (Table 1). These QTL effects had overlapping positions. Two independent QTLs were assigned to unhardened plants on chromosomes 1B and 7B and eleven QTLs were identified in cold-acclimated seedlings on chromosomes 7A, 1B, 2B, 6B, 7B, 3R, 5R, and 6R. Fifteen of these effects had a positive additive effect referred to *M. nivale* tolerant parental cv. ‘Hewo’. This effect was dedicated only to cv. ‘Hewo’ for all QTLs identified in the 1B linkage group in unhardened plants as well as in the 7A, 7B, and 3R linkage groups in cold-hardened plants (Table 1). Five candidate genes were identified, located on all the chromosomes listed above and shown in Table 2.

### 3.1. Phenotypic and QTLs Evaluation Associated with the Seedlings Susceptibility to M. nivale Infection (P Index) and Quantum Efficiency of Photosynthesis (Qy)

During all experimental seasons, the degree of the seedling’s damage after *M. nivale* inoculation was analyzed only for cold-hardened plants, since without hardening, all seedlings, irrespective of genotype, did not survive after mycelial infection. In the first year of the experiment, the mean P index value was high (P_2011_ = 61) and was similar to the mean value observed in the third year of the experiment. In the second year, the mean P index value was the lowest (P_2012_ = 41) among all years of research (Table S1). A positive correlation was found between P_2012_ and P_2013_ values as well as between P indexes of the individual experimental season and the mean P index of three years (Table S2). The highest positive correlation (1.00) was observed between P_2012_ and Qy_2012_ values (Table 3).

**Figure 1:**
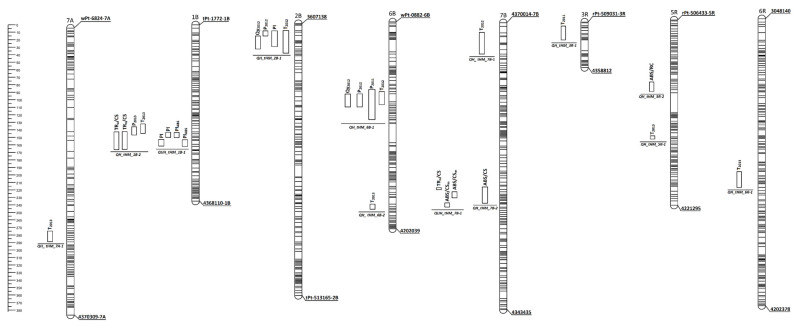
Interval map (cm) for chromosomes 7A, 1B, 2B, 6B, 7B, 3R, 5R, and 6R of ‘Hewo’ x ‘Magnat’ DH lines mapping population of winter triticale (x *Triticosecale*) with QTLs associated with: (1) the seedling damage caused by the *M. nivale* infection (P_2011_, P_2012_, and P_2013_ index); (2) the *M. nivale* infection tolerance (T_2011_, T_2012_, and T_2013_ index); (3) photosynthesis quantum efficiency (Qy_2012_) as well as (4) chlorophyll *a* fluorescence parameters, identified in unhardened and cold-hardened seedlings. All QTLs were identified by the SMA method. Black lines show markers identified by CIM method.

Four QTL effects associated with the P index (one for P_2011_, two for P_2012_, and one for P_2013_) and two for Qy_2012_ were assigned to three QTL regions on wheat linkage groups 1B, 2B, and 6B (Table 1, Figure 1). On chromosome 1B, QTL *QH_tHM_1B-2* containing effect for P_2012_ explained 11.12% of the trait phenotypic variability with LOD value 4.4 and had negative allele effect referring to cv. ‘Magnat’ (Table 1). On chromosome 2B, QTL *QH_tHM_2B-1* was related to P_2012_ and Qy_2012_ and explained up to 11.18% of the phenotypic trait variation with positive allele effect referred to cv. ‘Hewo’ and LOD value 3.1 for both traits (Table 1).

*Locus QH_tHM_6B-1*, identified on chromosome 6B contained QTL effects for P_2011_, P_2012_ and Qy_2012_ covered almost the same region on chromosome 6B between 69.9 and 110.6 cm (Table 1, Figure 1). The LOD value achieved up to 3.8 for P_2012_ trait and explained 10.94–12.06% of the phenotypic variation depending on the trait (Table 1). LOD peak was positioned at the same DArT-seq marker *3620975* and positive allele effect referred to cv. ‘Hewo’ for P_2012_ and Qy_2012_ (Table 1). At the same time, it was the season of the lowest average infestation P_2012_ of plants and the highest correlation between P index and Qy (Table S1, Table 3).

### 3.2. Phenotypic and QTLs Evaluation Associated with the Seedlings Tolerance to M. nivale Infection

Nine QTL effects associated with seedlings tolerance were identified on wheat: 7A, 1B, 2B, 6B, and 7B and rye: 3R, 5R, and 6R chromosomes, and assigned to nine QTLs (Table 1, Figure 1). Three of those effects were co-located with other effects related to other measured traits under separate QTL regions (Table 1). Among them, *QH_ tHM_7B-1* had a maximal LOD value (5.5). For the *M. nivale* tolerance in the first experimental season (T_2011_) only one locus was found (*QH_tHM_3R-1*) on chromosome 3R. It explained 14.12% of the trait phenotypic variability with LOD value 4.5 and had a positive allele effect referring to cv. ‘Hewo’ (Table 1). Another three QTLs, identified on chromosomes 2B, 6B, and 7B were associated with the second tolerance test results (T_2012_). They explained from 9.31% to 11.42% of the phenotypic variation and had LOD value ≥ 3. Two of them had an additive positive effect from cv. ‘Hewo’ (Table 1). QTLs assigned to T_2013_ were the most numerous–five, and covered regions on 7A, 1B, and 6B as well as 5R and 6R chromosomes. They explained from 5.60% to 15.81% of the phenotypic variation and had LOD value from 3.1 to 5.5. Two of them had an additive positive effect from cv. ‘Hewo’: *QH_tHM_1B-2* and *QH_ tHM_7A-1*. Only two QTLs associated with plant tolerance shared the same chromosome: *QH_tHM_6B-1* and *QH_tHM_6B-2*; however, had a different position (Table 1, Figure 1).

### 3.3. Phenotypic and QTLs Evaluation Associated with Chlorophyll Fluorescence Parameters

All measured Chl-a parameters were divided into three groups: (a) phenomenological energy fluxes calculated for the area of photosynthetic sample (CS) at t = 0, (b) parameters calculated per excided leaf-cross section (CS_m_), and (c) energy fluxes for single PSII reactions centers (RC). All the above parameters were measured in cold-hardened and unhardened plants.

The mean value of phenomenological energy fluxes (CS) group: TR_o_/CS, ET_o_/CS, DI_o_/CS, and ABS/CS was higher in cold-hardened seedlings than for unhardened plants; the highest difference was observed for ABS/CS (Table S1). The highest positive correlation (0.96) was found between ABS/CS and DI_o_/CS values measured in cold-hardened plants (Table S3). Contrarily, the highest negative correlation (−0.91) was found between DI_o_/CS and TR_o_/CS_m_ values measured in cold-hardened plants (Table S3).

The QTL regions within the group of phenomenological energy fluxes parameters were identified only for TR_o_/CS and ABS/CS traits (Table 1). On chromosome 1B, locus *QH_QH_tHM_1B-2* was found in cold-hardened seedlings; it explained 10.22% of the trait variation with LOD value 3.1. Negative allele effect of this locus referred to cv. ‘Magnat’. On chromosome 7B, QTL was identified for ABS/CS trait (*QH_tHM_7B-2*) in cold-hardened plants; it explained 13.28% of the trait variation with LOD value 3.8. Positive allele effect of this locus referred to cv. ‘Hewo’ (Table 1).

The mean value of the parameters calculated per excited leaf cross-section (CS_m_) was higher in unhardened plants, except for DI_o_/CS_m_, which was higher in plants after cold treatment (Table S1). The highest correlation within CS_m_ parameters was found between TR_o_/CS_m_ and ABS/CS_m_ values (0.95) measured in unhardened plants (Table S3). Within all measured parameters from all groups, the highest correlation was observed between TR_o_/CS_m_ and DI_o_/CS (−0.91) values as well as TR_o_/CS_m_ and PI (0.87) values measured in plants after cold treatment (Table S3).

One QTL effect was identified only for ABS/CS_m_ parameter and assigned to *QUH_tHM_7B-1* QTL on wheat chromosome 7B for unhardened plants. It explained 12.31–12.77 % of phenotypic variation with LOD value up to 3.7 and negative allele effect referred to cv. ‘Magnat’ (Table 1).

The mean value of parameters from energy fluxes for the single PSII reaction center (RC) group was higher in unhardened plants than in cold-hardened plants, apart from DI_o_/RC values being the same for both types of plants treatment (Table S1). The highest correlation was observed between ABS/RC and ABS/CS (0.87) as well as ABS/RC and PI (−0.90) measured in cold-hardened plants (Table S3). The most important QTL region was found for ABS/RC on chromosome 5R: locus *QH_tHM_5R-2* explained 15.45% of phenotypic variation with LOD value 4.5 and positive allele effect referred to cv. ‘Hewo’ (Table 1).

The mean value of the PI group was higher in unhardened plants than in cold-hardened (Table S1). One QTL region *QUH_tHM_1B-1* for these traits was found only on chromosome 1B (containing 4 QTL effects) for unhardened plants as well as one effect on chromosome 2B for cold-hardened ones assigned to *QH_tHM_2B-1* (Table 1). A positive allele effect of this loci referred to cv. ‘Hewo’ for unhardened seedlings, while for cold-hardened plants, a negative effect referred to cv. ‘Magnat’ was observed. Those loci explained 10.65–19.67% of phenotypic variation with LOD value up to 5.7 for *QUH_tHM_1B-1* (Table 1).

### 3.4. Comparison of Identified QTL Regions

For chromosomes 7A, 3R, and 6R, only one QTL was found, associated with *M. nivale* tolerance in cold-hardened plants (T_2013_, T_2011_, and T_2013_, respectively). On chromosomes, 6B, 7B, and 5R, the QTL regions identified for infection tolerance (T_2013_, T_2012_, and T_2013_, respectively) shared the linkage group with QTLs located for other traits but had a different distant cm position.

In contrast, among other identified genome regions, common QTLs for more than one trait were observed. Two QTLs containing five QTL effects were found on chromosome 1B, associated with the *M. nivale* tolerance T_2013_, the plant damage index caused by the infection P_2013_ and TR_o_/CS trait in cold-hardened seedlings as well as PI, and PI_ABS_ traits in unhardened seedlings (Table 1, Figure 1. All of these QTLs were located between 128.6 cm and 160.8 cm with the LOD value up to 5.7 for PI and PI_ABS_ effects on *QUH_tHM_1B-1*. Effects for T_2013_ and P_2013_ assigned to *QH_tHM_1B-2* QTL shared the same peak marker *4340874* as well as flanking marker *3619131.* Next, the same peak marker *3604249* was found for three QTL effects for TR_o_/CS,PI, and PI_ABS_ assigned to two QTLs. Moreover, the same peak marker *4372036* was found for three effects for TR_o_/CS, PI, and PI_ABS_ located on two QTLs.

On chromosome 2B, four QTL effects were assigned to QTL *QH_tHM_2B-1* and located between 9.3 cm and 37.5 cm, associated with *M. nivale* tolerance T_2012_, plant damage index caused by the infection P_2012_ and traits Qy_2012_, PI, exclusively in cold-hardened seedlings (Table 1, Figure 1). The phenotypic variation of those loci was between 10.43% and 11.42% with the LOD value up to 3.5 for the *PI* effect. Traits T_2012_, P_2012_, and PI shared the same flanking marker *4341334*, while P_2012_ and Qy_2012_ traits shared the same flanking marker *4344975.* QTL effects for T_2012_ and Qy_2012_ traits had the same peak marker *4360063*. The additive effect referred to ‘Hewo’ for P_2012_ and Qy_2012_ traits in cold-hardened plants (Table 1).

Four common QTL effects assigned to *QH_tHM_6B-1* were found on chromosome 6B, located between 93.9 cm and 128.2 cm and associated with T_2012_, P_2011_, P_2012_, and Qy_2012_ traits, all in cold-hardened seedlings (Table 1, Figure 1). Marker *3620975* had the same peak position for effects found for T_2012_, P_2012_, and Qy_2012_. Like on chromosome 2B, the additive effect referred to ‘Hewo’ for P_2012_ and Qy_2012_ traits in cold-hardened plants (Table 1). On chromosome 7B, four effects assigned to three different QTLs were common and identified for chlorophyll fluorescence parameters TR_o_/CS and ABS/CS_m_ in unhardened plants as well as ABS/CS in cold-hardened ones between 227.7 and 258.3 cm (Table 1, Figure 1).

### 3.5. Candidate Genes within the Main QTLs

The candidate genes identified for *M. nivale* tolerance of cold-hardened seedlings include those coding sterol 3-beta-glucosyltransferase UGT80A2-like (LOC123043096) in locus *QH_tHM_2B-1* associated with PI, Qy_2012_, P_2012_, and T_2012_ traits as well as transcription factor NAI1-like (LOC123094224) and flavonol3-sulfotransferase-like (LOC123098819) in locus *QH_tHM_5R-1* associated with ABS/RC trait (Table 2).

The candidate genes identified in unhardened plants were those coding protein DMP3-like (LOC119342110) and uncharacterized LOC123162005in locus *QUH__tHM_7B-1* identified for ABS/Cs_m_ and TR_O_/CS traits (Table 2).

## 4. Discussion

In this study, we presented the results of winter triticale seedlings damage caused by *M. nivale* infection (susceptibility/tolerance level), photosynthesis quantum efficiency and chlorophyll *a* fluorescence measurements as well as QTL regions associated with analyzed traits together with candidate genes. As previously described, the analysis of chlorophyll *a* fluorescence can be used to estimate plant tolerance to different stress conditions e.g., drought, freezing, and high temperature as well as fungal infection [49,50,51,52,53,54,55,56]. Additionally, the JIP test can be easily used as an indicator of the activity of the photosynthetic apparatus [57]. In the present study, chlorophyll *a* fluorescence parameters were measured in cold-hardened plants in comparison to unhardened plants. Based on our previous studies performed on seedlings of different winter triticale genotypes, it can be assumed that cold-hardening strongly increases *M. nivale* tolerance and it is significantly connected with the chlorophyll *a* parameters value [14,17,18,20]. That statement correlates with P index values observed in presented studies, especially during the second year of the experiment. It was confirmed before [18,20] that triticale reveals genotype-dependent cold-primed tolerance to *M. nivale* infection. In the present work, the highest average values of phenomenological energy flux parameters (CS) were observed in plants after cold treatment in relation to unhardened ones. The correlation between the P index and parameters from the CS group was observed in cold-hardened plants. In contrast, parameters from CSm and RC groups (parameters calculated per excited leaf-cross section and energy fluxes for single PSII reaction centers, respectively) were higher for unhardened plants.

In cold-acclimated plants, analysis of QTL regions revealed *loci* associated with the level of *M. nivale* tolerance/susceptibility and chlorophyll fluorescence parameters. QTLs related to photosynthesis parameters were located on 1B (TR_o_/CS), 2B (PI and Qy), 6B (Qy), 7B (ABS/CS), and 5R (ABS/RC) chromosomes. Simultaneously, nine QTL effects assigned to nine different QTL regions associated with seedlings tolerance were identified on 7A, 1B, 2B, 6B, 7B, 3R, 5R, and 6R chromosomes as well as four QTL effects assigned to three QTLs associated with P index on wheat linkage groups 1B, 2B, and 6B. All above loci were unique for cold-hardened plants, thus associated with their cold-acclimation processes.

Moreover, in the present study, QTLs with the similar position for seedlings, *M. nivale* tolerance/susceptibility and photosynthesis traits were identified. Four common QTL effects assigned to one QTL region were found on chromosome 1B, associated with the tolerance T_2013_, the plant damage index caused by the infection P_2013_, and TR_o_/CS trait in cold-hardened seedlings. The additive effect for T_2013_ trait referred to cv. ‘Hewo’. On chromosome 2B, four common QTL effects assigned to one locus were associated with T_2012_, P_2012_ as well as Qy_2012_ and PI traits, exclusively in cold-hardened seedlings. The additive effect referred to cv. ‘Hewo’ for P_2012_ and Qy_2012_ traits in cold-hardened plants. Finally, four QTL effects assigned to one locus were found on chromosome 6B, associated with T_2012_, P_2011_, P_2012_, and Qy_2012_ traits, all in cold-hardened seedlings. As with chromosome 2B, the additive effect referred to cv. ‘Hewo’ for P_2012_ and Qy_2012_ traits in cold-hardened plants. The above results indicate that TR_o_/CS, Qy_2012_, and PI traits loci could be potential tolerance level markers because they shared genome regions with the P and T indexes traits.

In contrast, for chromosomes 7A, 3R, and 6R only one QTL effect in one locus was found to be associated with *M. nivale* tolerance in cold-hardened plants. On chromosomes, 6B, 7B, and 5R, the QTL regions identified for infection tolerance shared the linkage group with QTLs located for other traits but had a different, distant cm position. Those loci could be thus considered as unique markers of the level of tolerance, not related with other measured parameters.

The most important candidate genes identified for *M. nivale* tolerance of cold-hardened seedlings include those coding: (1) sterol 3-beta-glucosyltransferase UGT80A2-like found on QH_tHM_2B-1 and (2) transcription factor NAI1-like, transcript variant X2 and flavonol3-sulfotransferase-like on QH_tHM_5R-1 (Table 2). Such results suggest that after seedlings’ cold-acclimation, two transferases, as well as DNA-binding transcription factor could be assumed to be involved in preceding biotic stress tolerance.

On chromosome 6B, one locus containing QTL effects associated with P index, T index, and Qy parameters were identified. This chromosome has been previously described as a chromosome containing many QTL regions assigned with multiple chlorophyll *a* parameters [57,58,59,60]. In our study, significant QTL regions identified in the rye genome were found on chromosome 5R, one locus was associated with traits from RC group for hardened plants, covering the chromosome region between 82.5 cm and 97.1 cm together with QTL for T index located between 155.1 cm and 161.3 cm (Table 1). The chromosome 5R has been previously described as a source of QTL regions associated with multiply Chl-*a* traits in the rye [60,61,62] and also triticale [14,50]. The number of DRA (disease resistance associated) genes was intermediate for 5R (242–255) [63] high-quality rye genome assembly.

Chromosomes from the B genome group have been previously described as chromosomes carrying loci associated with multiple chlorophyll-related parameters [14,55,64,65]. Moreover, in our study, QTL effects have common regions for different traits on chromosome 1B, which can be evidence of the importance of this chromosome for controlling those traits. Zhang et al. [66] identified loci on that chromosome associated with chlorophyll fluorescence values in wheat. Testing wheat under drought stress, Ilyas et al. [65] identified a major QTL region for chlorophyll content with an LOD score of 5.5 on wheat chromosome 1B. Yang et al. [64] found wheat drought tolerance *loci* to be associated with parameters of chlorophyll fluorescence kinetics (PCFKs) on chromosome 1B. Similarly, Dyda et al. [14] reported QTL regions on chromosome 1B related to leaf damage and systemic *Fv/Fm* after infection with two different *M. nivale* isolates. On chromosome 2B, the QTL region associated with *Fv/Fm* has been identified by other authors [57]. In our study, chromosome 7B revealed the highest number of QTL effects associated with different Chl-*a* parameters. Similarly, two QTL for Chl-*a* parameters from the phenomenological energy flux (CS) group were found by Czyczyło-Mysza et al. [57]. Ilyas et al. [65] also reported significant QTL regions on 7B chromosome with an LOD value up to 5.51 for one locus associated with total chlorophyll content in wheat under drought stress.

For several flanking markers important in our analysis, similar associations were also found in other studies. For example, the marker *wPt-7887* flanked important QTL region for wheat leaf rust and grain yield [67] studies. Another marker *wPt-2725* flanked important QTL region for wheat resistance and susceptibility to *Septoria tritici* blotch [68]. Next, *Pt-1723* (87.6) and *wPt-9195* (88.3) markers flanked Tan spot severity genome region in adult wheat plants identified by Shankar et al. [69].

As suggested by other authors, partial *M. nivale* tolerance could be found even in unhardened plants [70]. Furthermore, in our study, common QTL effects assigned to one locus associated with chlorophyll *a* parameters and tolerance/susceptibility traits were found in unhardened triticale seedlings on chromosome 1B (PI and PI_ABS_ traits). Moreover, the same peak markers were found for QTL effects for TR_o_/CS, *PI* and PI_ABS_ assigned to two loci (*QUH_tHM_1B-1* and *QH_tHM_1B-2*). On chromosome 7B, four common QTL effects assigned to two loci were identified for chlorophyll fluorescence parameters TR_o_/CS and ABS/CS_m_ in unhardened plants as well as ABS/CS in cold-hardened ones. The candidate gene was identified in unhardened plants and DMP3-like protein was identified in chromosome 7B. However, both the number of identified QTLs and candidate genes were lower in unhardened plants than in cold-hardened ones.

In summary, the most important QTLs associated with winter triticale seedlings’ cold-induced tolerance to *M. nivale* infection were located on chromosomes 7A, 6B, 3R, 5R, and 6R. Within those genome regions, the important candidate genes coding two transferases and DNA-binding transcription factors were found. QTLs for TRo/CS and PI traits were identified both in unhardened and cold-hardened seedlings. The results indicate that TR_o_/CS, Qy, and PI traits loci could be potential tolerance level markers because they shared genome regions with the P and T indexes traits.

## Figures and Tables

**Table 1:** The characteristics of the most significant QTLs associated with: (1) the seedling damage caused by the *M. nivale* infection (P_2011_, P_2012_ and P_2013_ index); (2) the *M. nivale* infection tolerance (T_2011_, T_2012_ and T_2013_ index); (3) photosynthesis quantum efficiency (Qy_2012_) as well as (4) chlorophyll *a* fluorescence parameters, identified in unhardened and cold-hardened winter triticale seedlings of the DH ‘Hewo’ x ‘Magnat’ lines mapping population (^a^—an identifiable region of the QTL defined by the first and last marker of the QTL region; ^b^—the percentage of the phenotypic variance explained by the QTL-R^2^ (%); ^c^—additive effect (Add) referred to H—parental cv. ‘Hewo’ or M—parental cv. ‘Magnat’).

QTL Name	Trait	Flanking Markers ^a^ (Position in cm)	LOD	LOD Max. Position	Marker Closest to the LOD Peak	R^2^ (%) ^b^	Add ^c^	Favorable Allele
**Unhardened seedlings**								
*QUH_tHM_1B-1*	**PI**	*3619131*–*wPt-5899-1B * (144.1–150.0)	5.7	146.7	*3604249*	19.67	0.29	H (+)
		*4371570*–*4345821* (154.1–160.8)	4.2	155.5	*4372036*	16.82	0.27	
	**PI_ABS_**	*3619131*–*wPt-5899-1B * (144.1–150.0)	5.7	146.7	*3604249*	19.67	0.29	
		*4371570*–*4345821* (154.1–160.8)	4.2	155.5	*4372036*	16.82	0.27	
*QUH_tHM_7B-1*	**ABS/CS_m_**	*4342266*–*4204248* (230.8–238.1)	3.7	236.1	*4347086*	12.77	−60.81	M (−)
		*3618369*–*wPt-7887-7B * (252.5–258.3)	3.5	258.3	*wPt-7887-7B*	12.31	−59.79	
	**TR_o_/CS**	*4371643*–*4342266* (227.7–230.8)	4.5	227.7	*4371643*	19.59	12.26	H (+)
**Cold-hardened seedlings**								
*QH_tHM_1B-2*	**P_2013_**	*wPt-2725-1B*–*3619131 * (133.1–144.1)	4.4	139.2	*4340874*	11.12	−3.55	M (−)
	**TR_o_/CS**	*4340874–4345821* (139.2–160.8)	3.0	146.7	*3604249*	10.91	−5.55	
		*4340874–4345821* (139.2–160.8)	3.1	155.5	*4372036*	10.22	−5.65	
	**T_2013_**	*4203983–3619131* (128.6–144.1)	3.1	139.2	*4340874*	7.13	2.91	H (+)
*QH_tHM_2B-1*	**T_2012_**	*4341334–4200529* (9.3–37.5)	3.2	25.2	*4360063*	11.42	−2.71	M (−)
	**PI**	*4341334–4350667* (9.3–22.9)	3.5	16.1	*4344975*	10.65	−0.16	
	**P_2012_**	*4341334–4344975* (9.3–16.1)	3.1	11.6	*4349576*	10.43	2.49	H (+)
	**Qy_2012_**	*4344975–4342979* (16.1–34.2)	3.1	25.2	*4360063*	11.18	2.69	
*QH_tHM_6B-1*	**T_2012_**	*3620975–3619273* (96.9–110.6)	3.0	96.9	*3620975*	10.71	−2.72	M (−)
	**P_2011_**	*4357005–3040595-6B * (93.9–128.3)	3.6	115.3	*4204618*	11.34	−2.93	
	**P_2012_**	*3620975–3619273* (96.9–110.6)	3.8	96.9	*3620975*	10.94	2.74	H (+)
	**Qy_2012_**	*3620975–3619273* (96.9–110.6)	3.1	96.9	*3620975*	12.06	2.88	
*QH_tHM_6B-2*	**T_2013_**	*wPt-5480-6B–3609814 * (248.7–254.8)	4.2	252.8	*4204529*	11.63	−3.72	M (−)
*QH_tHM_7A-1*	**T_2013_**	*4366013–4364182-7A * (270.1–284.8)	5.5	273.9	*4345861*	15.81	4.23	H (+)
*QH_tHM_7B-1*	**T_2012_**	*4360568–4342014* (17.6–42.2)	3.0	29.6	*4350658*	9.31	2.52	H (+)
*QH_tHM_7B-2*	**ABS/CS**	*4343332–3618369* (242.1–252.5)	3.8	248.2	*4205202*	13.28	32.96	
*QH_tHM_3R-1*	**T_2011_**	*3603712–4373226* (6.8–22.7)	4.5	17.4	*4202139*	14.12	3.80	H (+)
*QH_tHM_5R-1*	**T_2013_**	*3611246–rPt-390144-5R * (155.1–161.3)	5.1	158.5	*4343941*	14.33	−4.40	M (−)
*QH_tHM_5R-2*	**ABS/RC**	*4356596–4373163* *(82.5–97.1)*	4.5	90.7	*3624321*	15.45	0.21	H (+)
*QH_tHM_6R-1*	**T_2013_**	*3621767–3618196* *(213.2–225.2)*	3.1	220.4	*3044945*	5.60	−2.62	M (−)

**Table 2:** In silico identification of candidate genes found within the significant QTL regions associated with the seedling damage caused by the *M. nivale* infection (P index), the *M. nivale* infection tolerance (T index), photosynthesis quantum efficiency (Qy_2012_) as well as chlorophyll *a* fluorescence parameters in winter triticale seedlings of the DH ‘Hewo’ x ‘Magnat’ lines mapping population.

QTL (Trait)	Marker/ Position	Gene Name	Position	Predicted Protein	Reference Organism	NCBI ID	Predicted Function
**Unhardened seedlings**							
*QUH__tHM_7B-1* (ABS/Csm, TR_O_/CS)	4204248 (flanking) Chr7B:672350251..672350320	TraesCS7B03G1075200	Chr7B:672349662..672350761 (+ strand)	Protein DMP3-like (LOC119342110)	*T. dicoccoides*	XM_037613949.1	Endomembrane system organization (GO: 0010256)
	4371643 (flanking/LOD) Chr7B:663773050..663773080	TraesCS7B03G1051500LC *	Chr7B:663772662..663773372 (− strand)	Uncharacterized LOC123162005	*T. aestivum*	XM_044579811.1	Unknown
**Cold-hardened seedlings**							
*QH_tHM_2B-1* (PI, Qy_2012_, P_2012_, T_2012_)	4344975 (LOD) Chr2B:9347050..9347062	TraesCS2B03G0035600LC *	Chr2B:9347033..9351375 (− strand)	Sterol 3-beta-glucosyltransferase UGT80A2-like (LOC123043096)	*T. aestivum*	XM_044465446.1	Lipid glycosylation (GO: 0030259); UDP-glycosyltransferase activity (GO: 0008194); carbohydrate metabolic process (GO: 0005975)
*QH_tHM_5R-1* (ABS/RC)	4356596 (flanking) Chr5R:835874349..835874381	SECCE5Rv1G0369560	Chr5R:835868920..835875125 (− strand)	Transcription factor NAI1-like (LOC123094224)	*T. aestivum*	XR_006445773.1	Protein dimerization activity (GO: 0046983); metal ion binding (GO: 0046872)

**Table 3:** Significant correlation between the seedling damage caused by the *M. nivale* infection and the selected traits evaluated in cold-hardened winter triticale seedlings of ‘Hewo’ x ‘Magnat’ DH lines.

Trait	Candidate Gene/Protein	Seedling Damage Caused by the *M. nivale* Infection				Tolerance to the *M. nivale* Infection			
		P_2011_	P_2012_	P_2013_	P_2011–2013_	T_2011_	T_2012_	T_2013_	T_2011–2013_
P_2011_					**0.51**				**−0.51**
P_2012_	Sterol 3-beta-glucosyltransferase UGT80A2-like			**0.32**	**0.57**			**−0.32**	**−0.57**
P_2013_					**0.78**				**−0.78**
T_2011_					**−0.51**				**0.51**
T_2012_	Sterol 3-beta-glucosyltransferase UGT80A2-like			**−0.32**	**−0.57**			**0.32**	**0.57**
T_2013_					**−0.78**				**0.78**
ABS/CS			0.28	0.2	0.26		−0.28	−0.2	−0.26
ABS/RC	Transcription factor NAI1-like, Flavonol3-sulfotransferase-like			0.23	0.22			−0.23	−0.22
PI	Sterol 3-beta-glucosyltransferase UGT80A2-like		−0.2	−0.24	−0.24		0.2	0.24	0.24
Tr_o_/CS			0.32				**−0.32**		
Qy_2012_	Sterol 3-beta-glucosyltransferase UGT80A2-like	0.97	1.00	0.32	0.57	−0.97	−1.00	−0.32	−0.57

## Data Availability

Authors confirm that all data, tables and figures in this manuscript are original.

## Supplementary Materials

The following are available online at https://www.mdpi.com/article/10.3390/plants10122678/s1, Table S1: Values distribution of the measured traits. W—the value of the Shapiro–Wilk normality test at *p* ≤ 0.05, Table S2: Value and direction of correlation between the levels of the seedling damage caused by the *M. nivale* infection (P indexes) in three independent experiments (seasons), Table S3: Value and direction of correlation between the values of the individual chlorophyll *a* fluorescence parameters for which significant QTL regions have been found.
