# Supplementary material for: Quantitative Trait Loci and Candidate Genes Associated with Cold-Acclimation and Microdochium nivale Tolerance/Susceptibility in Winter Triticale (x Triticosecale)
Source: Plants (Basel). 2021 Dec 6;10(12):2678. doi: 10.3390/plants10122678 (PMC8704164; doi:10.3390/plants10122678)
Supplement: Supplementary file 1 [file plants-10-02678-s001.zip › plants-1442326-supplementary.pdf]

Supplementary Materials

Table S1. Values distribution of the measured traits. W – the value of the Shapiro-Wilk normality test at $p \leq 0.05$.

Trait	Minimum – Maximum	Average ± SD	W*	Skewness	Kurtosis
P_{2011} hardened [%]	37.50 – 85.00	61.17 ± 9.36	0.989	−0.03	0.18
P_{2012} hardened [%]	25.00 – 63.33	41.41 ± 7.95	0.986	0.24	0.14
P_{2013} hardened [%]	29.17 – 81.67	61.22 ± 10.35	0.951	−0.73	0.27
T_{2011} hardened [%]	15.00 – 62.50	38.83 ± 9.36	0.988	0.03	0.18
T_{2012} hardened [%]	36.67 – 75.00	58.59 ± 7.95	0.983	−0.24	0.14
T_{2013} hardened [%]	18.33 – 70.83	38.78 ± 10.35	0.951	0.73	0.27
Qy_{2011} hardened	37.50 – 85.00	61.17 ± 9.36	0.988	−0.03	0.18
Qy_{2012} hardened	25.00 – 63.33	41.41 ± 7.95	0.986	0.24	0.14
PI unhardened	0.55 – 2.38	1.25 ± 0.36	0.981	0.48	0.47
PI hardened	0.36 – 2.03	1.11 ± 0.33	0.985	0.21	0.48
PI_{ABS} unhardened	0.55 – 2.38	1.25 ± 0.36	0.981	0.48	0.47
PI_{ABS} hardened	0.32 – 2.03	1.11 ± 0.32	0.983	0.20	0.68
ABS/CS unhardened	447.17 – 712.25	540.45 ± 54.77	0.874	0.35	0.75
ABS/CS hardened	531.33 – 759.50	307.92 ± 42.97	0.939	0.16	0.89
TR_o/CS unhardened	373.31 – 515.71	437.64 ± 25.98	0.951	0.65	0.23
TR_o/CS hardened	428.18 – 534.54	472.01 ± 16.59	0.971	0.61	0.99
ET_o/CS unhardened	144.80 – 271.29	194.93 ± 21.45	0.960	0.78	0.76
ET_o/CS hardened	187.29 – 235.84	219.21 ± 7.69	0.919	−0.33	0.69
DI_o/CS unhardened	69.47 – 230.16	102.81 ± 31.21	0.771	0.91	0.61
DI_o/CS hardened	89.31 – 240.54	135.91 ± 29.69	0.900	0.37	0.59
ABS/CS_m unhardened	2268.75 – 3339.00	2972.07 ± 195.93	0.951	−0.92	0.85
ABS/CS_m hardened	2072.75 – 3242.00	2836.24 ± 209.57	0.966	−0.75	0.15
TR_o/CS_m unhardened	1556.50 – 2808.00	2431.62 ± 229.80	0.927	−0.21	0.33
TR_o/CS_m hardened	1299.50 – 2703.00	2216.64 ± 253.03	0.962	−0.83	0.31
ET_o/CS_m unhardened	779.80 – 1492.50	1082.29 ± 135.90	0.989	0.34	0.11
ET_o/CS_m hardened	602.25 – 1360.33	1037.40 ± 140.71	0.988	−0.31	0.57
DI_o/CS_m unhardened	447.17 – 712.25	540.45 ± 54.77	0.874	0.35	0.73
DI_o/CS_m hardened	531.33 – 798.20	619.60 ± 54.16	0.928	0.10	0.53
RC/CS_m unhardened	466.46 – 1196.15	970.97 ± 147.14	0.955	−0.80	0.95
RC/CS_m hardened	446.86 – 1268.72	923.57 ± 143.02	0.979	−0.54	−0.13
ABS/RC unhardened	2.64 – 5.22	3.16 ± 0.43	0.832	0.25	0.27
ABS/RC hardened	2.59 – 3.74	3.07 ± 0.24	0.975	0.59	0.42
TR_o/RC unhardened	2.21 – 3.49	2.56 ± 0.22	0.919	0.35	0.59
TR_o/RC hardened	2.14 – 2.65	2.40 ± 0.11	0.989	0.04	−0.33
ET_o/RC unhardened	0.95 – 1.99	1.14 ± 0.14	0.717	0.18	0.35
ET_o/RC hardened	1.03 – 1.19	1.11 ± 0.04	0.983	0.10	−0.67
DI_o/RC unhardened	0.40 – 1.72	0.61 ± 0.22	0.724	0.57	0.54
DI_o/RC hardened	0.44 – 1.51	0.61 ± 0.17	0.565	0.84	0.64

Table S2. Value and direction of correlation between the levels of the seedling damage caused by the *M. nivale* infection (P indexes) in three independent experiments (seasons).

	P_{2012}	P_{2013}	$P_{2011–2013}$	$P_{2012–2013}$
P_{2011}	−0.15	0.07	**0.51**	−0.03
P_{2012}		**0.32**	**0.57**	**0.75**
P_{2013}			**0.78**	**0.86**
$P_{2011–2013}$				**0.84**

Table S3. Value and direction of correlation between the values of the individual chlorophyll *a* fluorescence parameters, for which significant QTL regions have been found.

	Qy_{2012}	PI_UH	PI_H	TR_0/CS_UH	TR_0/CS_H	ABS/CS_H	ABS/CS_m_UH	PI_{ABS}_UH
**PI_UH**	0.11							
**PI_H**	−0.19	0,35						
**TR_0/CS_UH**	0.23	−0.47	−0.32					
**TR_0/CS_H**	0.31	−0.19	−0.71	0.31				
**ABS/CS_H**	0.27	−0.88	−0.27	0.87	0.35			
**ABS/CS_m_UH**	0.03	0.74	0.37	−0.28	−0.30	−0.36		
**PI_{ABS}_UH**	0.11	1.00	0.35	−0.47	−0.19	−0.27	0.74	
**ABS/RC_H**	0.16	−0.34	−0.90	0.31	0.72	0.87	−0.36	−0.34
